# NEDD4L is a promoter for angiogenesis and cell proliferation in human umbilical vein endothelial cells

**DOI:** 10.1111/jcmm.18233

**Published:** 2024-03-25

**Authors:** Binghong Liu, Fei Song, Xiaoxia Zhou, Chan Wu, Huizhu Huang, Weiyin Wu, Gang Li, Yan Wang

**Affiliations:** ^1^ Medical College Guangxi University Nanning Guangxi China; ^2^ Xiamen Cardiovascular Hospital of Xiamen University, School of Medicine Xiamen University Xiamen Fujian China

**Keywords:** angiogenesis, cell migration, cell proliferation, NEDD4L

## Abstract

Dysregulated angiogenesis leads to neovascularization, which can promote or exacerbate various diseases. Previous studies have proved that NEDD4L plays an important role in hypertension and atherosclerosis. Hence, we hypothesized that NEDD4L may be a critical regulator of endothelial cell (EC) function. This study aimed to define the role of NEDD4L in regulating EC angiogenesis and elucidate their underlying mechanisms. Loss‐ and gain‐of‐function of NEDD4L detected the angiogenesis and mobility role in human umbilical vein endothelial cells (HUVECs) using Matrigel tube formation assay, cell proliferation and migration. Pharmacological pathway inhibitors and western blot were used to determine the underlying mechanism of NEDD4L‐regulated endothelial functions. Knockdown of NEDD4L suppressed tube formation, cell proliferation and cell migration in HUVECs, whereas NEDD4L overexpression promoted these functions. Moreover, NEDD4L‐regulated angiogenesis and cell progression are associated with the phosphorylation of Akt, Erk1/2 and eNOS and the expression of VEGFR2 and cyclin D1 and D3. Mechanically, further evidence was confirmed by using Akt blocker MK‐2206, Erk1/2 blocker U0126 and eNOS blocker L‐NAME. Overexpression NEDD4L‐promoted angiogenesis, cell migration and cell proliferation were restrained by these inhibitors. In addition, overexpression NEDD4L‐promoted cell cycle‐related proteins cyclin D1 and D3 were also suppressed by Akt blocker MK‐2206, Erk1/2 blocker U0126 and eNOS blocker L‐NAME. Our results demonstrated a novel finding that NEDD4L promotes angiogenesis and cell progression by regulating the Akt/Erk/eNOS pathways.

## 1 INTRODUCTION

Angiogenesis is fundamental to the establishment of a functional vasculature, which is essential for embryonic development, postnatal inflammatory responses and tissue regeneration.^1^ Dysregulated angiogenesis leads to neovascularization, and promotes or exacerbates diabetic retinopathy, age‐related macular degeneration and cancer growth.^2^ Pro‐ and anti‐angiogenic factors keep a dynamic balance under physiological conditions. Disturbance of this balance can lead to abnormal angiogenesis, leading to various diseases including cancer, diabetic retinopathy, vascular malformations and delayed wound healing.^3^ Intensive efforts have been made in the past decades to develop therapeutic strategies to promote the revascularization of ischemic tissues or inhibit angiogenesis in cancer, ocular, joint or skin disorders. However, clinical trials testing the pro‐angiogenic potential of vascular endothelial growth factor (VEGF) and fibroblast growth factor (FGF) have not yielded the expected results.^4^ Therefore, a novel molecule capable of regulating the growth of distal capillaries and proximal collateral conduit vessels may be an exciting approach to developing therapeutic dysregulated angiogenesis.

The ubiquitin to substrate proteins is called ubiquitination, which is a low molecular weight protein with highly conserved evolution.^5^ Ubiquitination marks the target proteins, allowing them to be recognized and degraded by the proteasome. Hence, as an important and common form of protein post‐translational modification, ubiquitination modifications usually occur on lysine residues and thereby regulate various cellular pathways.^6^, ^7^ The whole process of ubiquitination is formed by a ubiquitin‐proteasome system (UPS), which is mainly composed of ubiquitin, ubiquitin‐activating enzyme (E1), ubiquitin cross‐linking enzyme (E2), ubiquitin ligase (E3), deubiquitinating enzymes (DUBs) and 26S proteasome.^8^ The Neural precursor cell‐expressed developmentally downregulated 4 (NEDD4) family is one of the most essential groups of E3 ligase,^9^ which includes neuronal precursor cell expression developmentally downregulated 4‐1 (RPF1), neuronal precursor cell expression developmentally downregulated 4‐like (Nedd4L/Nedd4‐2), ITCH/atropine‐1 interacting protein 4 (AIP4), etc.^10^, ^11^, ^12^ NEDD4L (also known as Nedd4‐2) is a HECT (homologous to E6AP C terminus) E3 ubiquitin ligase of the highly conserved NEDD4 family. NEDD4L regulates a number of proteins that participate in epithelial Na+ channel regulation, DNA repair, autophagy and antiviral immunity.^13^, ^14^, ^15^, ^16^ Many previous studies established the association of NEDD4L with cardiovascular disease. A series of clinical and basic research have demonstrated that NEDD4L is associated with hypertension.^17^, ^18^, ^19^, ^20^ Our recent work has proved that NEDD4L participated in miR‐30a‐attenuated atherosclerosis.^21^ Nonetheless, the role of NEDD4L on endothelial cells has yet to be fully illustrated.

In the present study, we applied a series of loss‐ and gain‐function of NEDD4L to explore the role of anti‐ and pro‐angiogenic, cell proliferation and migration in the human umbilical vein endothelial cells (HUVECs). The result showed that the knockdown of NEDD4L suppressed the angiogenesis, cell migration and proliferation in HUVECs. However, overexpression of NEDD4L exhibited a pro‐angiogenic effect. Moreover, the Akt/Erk/eNOS pathway is involved in NEDD4L‐regulated these functions, which was also validated by pharmaceutical using Akt blocker MK‐2206, Erk1/2 blocker U0126 and eNOS blocker L‐NAME.

## 2 METHODS

### 2.1 Materials and reagents

Endothelial Cell Medium (ECM, Cat#: 1001) was obtained from ScienCell (Carlsbad, CA, USA). The cell culture dishes and plates, 6.5 mm Transwell® with 8.0 μm Pore (Cat#: 3422) and the Matrigel Matrix Basement Membrane (Cat#: 356230) were bought from Corning Inc. (Corning, NY, USA). The signalling pathway blockers MK‐2206 dihydrochloride (Cat#: HY‐10358), U0126‐EtOH (Cat#: HY‐12031) and L‐NAME hydrochloride (Cat#: HY‐18729A) were bought from MedChemExpress (MCE, New Jersey, USA). The primary antibodies, such as rabbit anti‐NEDD4L (Cat#: 4013), rabbit anti‐phospho‐Akt (Ser473) (Cat#: 4060S), rabbit anti‐Akt (Cat#: 9272), rabbit anti‐VEGF Receptor 2 (Cat#: 9698), rabbit anti‐phospho‐Erk1/2 (Cat#: #4370), rabbit anti‐Erk1/2 (Cat#: #4695), mouse anti‐Cyclin D3 (Cat#: 2936) and rabbit anti‐eNOS (Cat#: 32027), were from Cell Signaling Technology (Beverly, MA, United States). Anti‐phospho (S1177)‐eNOS (Cat#: ab215717), anti‐Cyclin D1 antibody‐C‐terminal (Cat#: ab134175) and the donkey anti‐rabbit IgG H&L (Alexa Fluor 488) (Cat#: ab150065) were purchased from Abcam (USA). The Phospho‐Histone H3‐S10 Rabbit antibody (Cat#: AP0840) was bought from ABclonal Technology (Wuhan, China). The polyvinylidene fluoride (PVDF) membranes were the products of Millipore (Billerica, MA, USA). Sodium Dodecyl Sulfate (SDS) and bovine serum albumin (BSA) were purchased from Sigma‐Aldrich (St. Louis, MO, USA). The Goat anti‐Rabbit IgG (H + L) Highly Cross‐Adsorbed Secondary Antibody Alexa Fluor™ 546 (Cat#: A‐11035) and Lipofectamine™ RNAiMAX Transfection Reagent (Cat#: 13778150) were bought from Invitrogen (USA). Control siRNA (Cat#: sc‐37007) and NEDD4L siRNA (Cat#: sc‐75894) were obtained from Santa Cruz (USA). The NEDD4L‐adenovirus (Order NO: HH202111‐2FJJY‐AD03) and control adenovirus (Order NO: HH202111‐2FJJY‐AD01) were bought from HANBIO (Shanghai, China).

### 2.2 Cell culture and siRNA transfection

Human umbilical vein endothelial cells (HUVECs) were purchased from the American Type Culture Collection (ATCC, USA). Cells were cultured in the ECM medium supplemented with 5% fetal bovine serum (FBS), 1% ECGs and 1% penicillin/streptomycin (ScienCell, USA) at 37°C in a CO_2_ incubator. After being transferred to 6‐well plates overnight, cells were co‐transfected with Lipofectamine RNAi MAX and NEDD4L‐siRNA, or control‐siRNA in OPTI‐MEM medium (Cat#: 31985070, Gibco, USA) for about 60 h. After that, cells were applied to detect the following tube formation assay, Transwell assay, immunostaining and western blotting assay.

### 2.3 Adenovirus infection

To detect the effect of overexpression of NEDD4L on HUVECs, we ordered the control and NEDD4L overexpressed adenovirus from HANBIO technology (Shanghai, China). Cells were infected with NEDD4L‐adenovirus and control adenovirus in ECM medium for 48 h. After infection, HUVECs were detected with tube formation assay, Transwell assay and immunostaining, as well as western blotting assay.

To examine the involved signalling pathway, cells were treated with Akt inhibitor MK‐2206, Erk1/2 inhibitor U0126 and eNOS inhibitor L‐NAME after being infected with adenovirus. Briefly, after being infected with these adenoviruses for 24 h, cells were treated with 100 nM MK‐2206 dihydrochloride (Cat#: HY‐10358, MedChemExpress, China), 20 μM U0126‐EtOH (Cat#: HY‐12031, MedChemExpress, China) and 100 μM L‐NAME hydrochloride (Cat#: HY‐18729A, MedChemExpress, China) for 24 h. Then, cells were collected for following detections, such as Matrigel tube formation assay, Transwell assay, western blotting and Phospho‐Histone H3 immunostaining.

### 2.4 Phospho‐Histone H3 immunostaining for cell proliferation assay

After treatment, HUVECs were fixed with 4% paraformaldehyde. Cells were then incubated with phospho‐Histone H3‐S10 antibody (Cat#: AP0840, ABclonal Technology, Wuhan, China) overnight at 4°C. Cells were then washed with phosphate‐buffered saline (PBS) three times. Then, cells were incubated with the secondary antibodies Donkey Anti‐Rabbit IgG H&L (Alexa Fluor® 488) (Cat#: ab150065, Abcam, USA) or Goat anti‐rabbit IgG (H + L) Highly Cross‐Adsorbed Secondary Antibody, Alexa Fluor™ 546 (Cat#: A‐11035, Invitrogen, USA). After being washed three times, cells were mounted with an antifading Mounting Medium with DAPI (Cat#: S2110, Solarbio, Beijing, China). Phospho‐Histone H3‐positive cells were captured with a Leica TCS SP5 II confocal microscope (Leica, Germany), and Phospho‐Histone H3‐positive cells were counted by Image J (NIH, USA).

### 2.5 Matrigel tube formation assay

The 96‐well cell culture plates were pre‐coated with growth‐factor–reduced Matrigel Matrix (50 μL, Corning, USA). HUVECs were suspended with FBS medium (1% FBS) and seeded in the pre‐coated plates. Six hours later, the tube formation ability of HUVECs was captured using phase‐contrast microscopy. The total tube length was analysed using Image J software with a plug‐in angiogenesis module (National Institutes of Health, USA).

### 2.6 Transwell migration assay

After treatment, the migration ability of the HUVECs was tested by using the Transwell assay as previously reported.^22^, ^23^, ^24^, ^25^ Briefly, a total of 2 × 10^4^ HUVECs (passage 3–8) were suspended in 200 μL ECM medium (0% FBS) and seeded in a modified Boyden chamber with an 8 μm‐pore polycarbonate membrane (Corning, USA) for 30 min. The lower chambers were placed with 600 μL ECM medium (2.5% FBS). Then, the plates were incubated at 37°C in a 5% CO_2_ incubator for 24 hours. After incubation, the chambers were washed with PBS and then fixed with 4% paraformaldehyde (Sigma‐Aldrich, USA). The membranes were then stained with 0.1% crystal violet for 20 minutes. After being washed, the non‐migrated cells on the upper membranes were wiped off with cotton swabs. The migrated cells were captured using phase‐contrast microscopy. The total number of migrated cells was determined by counting five fields in each well.

### 2.7 Western blot

After being treated with siRNA, adenovirus or inhibitors, HUVECs were suspended with RIPA lysis buffer (Cat#: P0013B, Beyotime, Beijing, China). Then, the BCA Protein Assay Kit (Cat#: P0010, Beyotime, Beijing, China) was applied to detect the protein concentration. After that, samples were boiled in 5 × SDS‐PAGE sample loading buffer (Cat#: P0015L, Beyotime, Beijing, China) at 100°C for 5 min. Samples (20 μg) were then loaded for SDS‐PAGE separation. Gels were transferred to polyvinylidene fluoride (PVDF) membranes. The membranes were blocked with 5% skimmed milk and blotted with primary antibodies against NEDD4L (Cat#: 4013, CST, USA), Akt (Cat#: 9272, CST, USA), phospho‐Akt (Ser473) (Cat: #4060, CST, USA), p44/42 MAPK (Erk1/2) (Cat: #4695, CST, USA), phospho‐p44/42 MAPK (Erk1/2) (Thr202/Tyr204) (Cat: #4370, CST, USA), VEGFR2 (Cat#: 9698, CST, USA), phospho‐eNOS (Cat#: ab215717, Abcam, USA), eNOS (Cat#: 32027, CST, USA), Cyclin D1 (Cat#: ab134175, Abcam, USA) and Cyclin D3 (Cat#: 2936, CST, USA) in a range of dilution factor from 1:500 to 1:2000 overnight at 4°C. The membranes were washed three times and then incubated with secondary antibodies for 1 h. The enhanced chemiluminescence reagent (Solarbio, Beijing, China) was applied for the detection of the blotting using a Bio‐Rad Chemiluminescence imaging system. Grey values were analysed with Image J software (NIH, USA).

### 2.8 Statistical analysis

Statistical significance was assessed using GraphPad Prism 9.0 to analyse data and produce graphs. Comparisons between the two groups were evaluated using Student's *t*‐test. One‐way anova analysis followed by Tukey's test for three or more groups. *p* Values of less than 0.05 were considered to indicate statistical significance. Data are expressed as mean ± SEM.

## 3 RESULTS

### 3.1 Knockdown of NEDD4L suppressed tube formation, cell migration and proliferation in HUVECs

**FIGURE 1:**
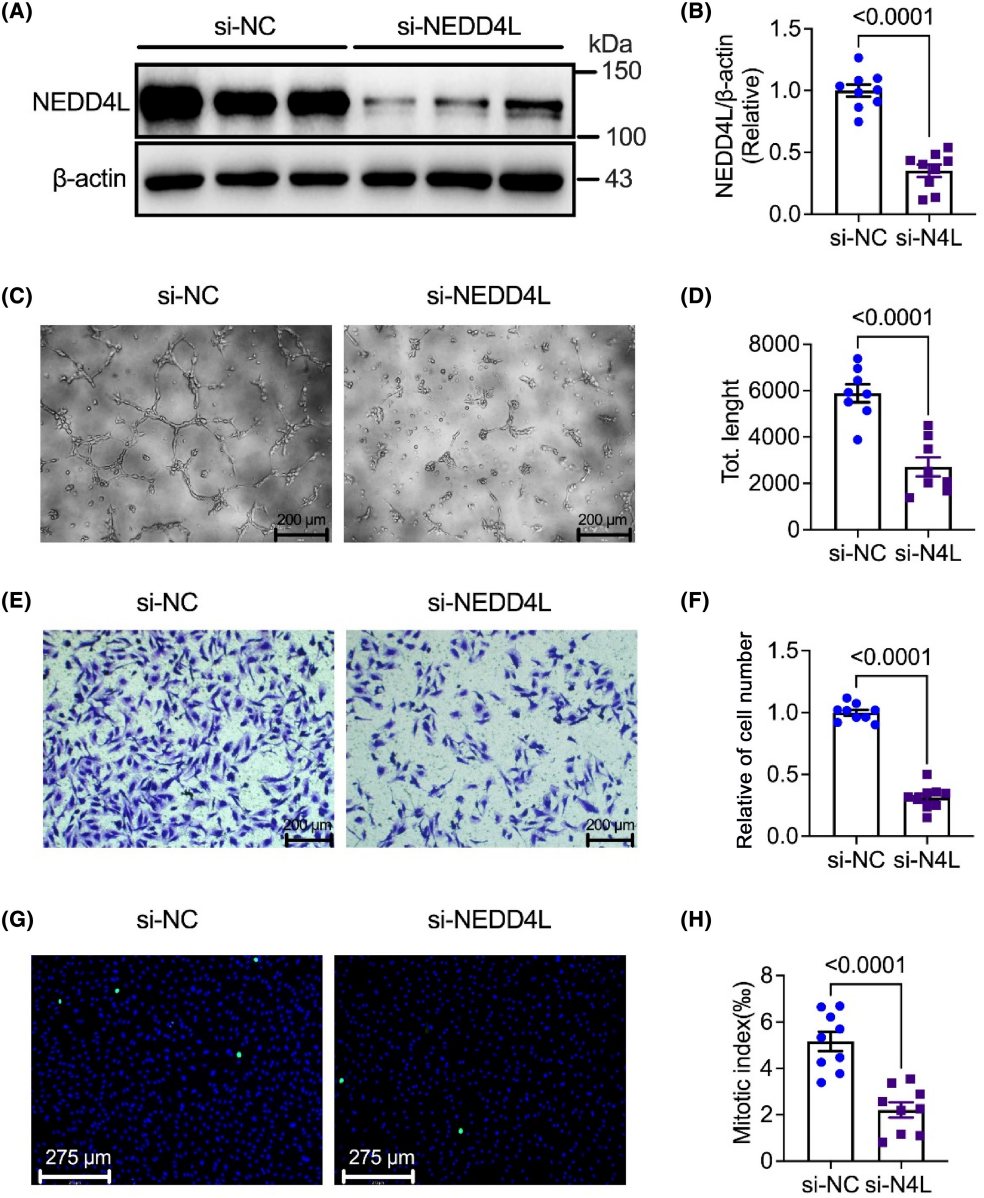
Deficiency NEDD4L suppressed angiogenesis, cell proliferation and migration in HUVECs. (A) Representative western blotting images of protein level of NEDD4L and β‐actin in HUVECs transfected with control siRNA (si‐NC) and NEDD4L siRNA (si‐NEDD4L). (B) Data analysis of relative expression of NEDD4L/β‐actin (*n* = 9 in each group). (C) Representative tube formation images with Matrigel matrix assay of HUVECs transfected with control siRNA (si‐NC) and NEDD4L siRNA (si‐NEDD4L). (D) Analysis of tube formation with ImageJ software revealed the total length (*n* = 8 in each group). (E) Representative transwell images of HUVECs transfected with control siRNA (si‐NC) and NEDD4L siRNA (si‐NEDD4L). (F) Data analysis of migrated cells of the transwell assay (*n* = 9 in each group). (G) Representative Phospho‐Histone H3 immunostaining images of HUVECs transfected with control siRNA (si‐NC) and NEDD4L siRNA (si‐NEDD4L). (H) Data analysis of proliferated cells of the Phospho‐Histone H3 staining (*n* = 9 in each group).

To determine whether NEDD4L regulates the angiogenic effect in HUVECs, we first knock down the NEDD4L expression by employing small interfering RNA (siRNA) in HUVECs. The knockdown efficiency was determined by western blotting which showed a significant decrease after transfected with NEDD4L siRNA (Figure 1A,B). We next examined the vasculogenic activity in HUVECs transfected with control siRNA and NEDD4L siRNA (Figure 1C,D) using the Matrigel tube formation assay. We established that angiogenic ability was significantly restrained by NEDD4L siRNA (Figure 1C,D). The motility role of deficiency of NEDD4L in HUVECs was assessed by transwell. The migrated cells were markedly suppressed in NEDD4L knockdown HUVECs (Figure 1E,F). The cell progression in the knockdown of NEDD4L on HUVECs was determined by pHH3 immunostaining in HUVECs. The pHH3‐positive cells exhibited a significant decrease in NEDD4L siRNA‐transfected HUVECs compared to control siRNA (Figure 1G,H). These data suggested that deficiency of NEDD4L has an anti‐angiogenic effect on HUVECs.

### 3.2 Overexpression NEDD4L exhibited a pro‐angiogenic effect in HUVECs

**FIGURE 2:**
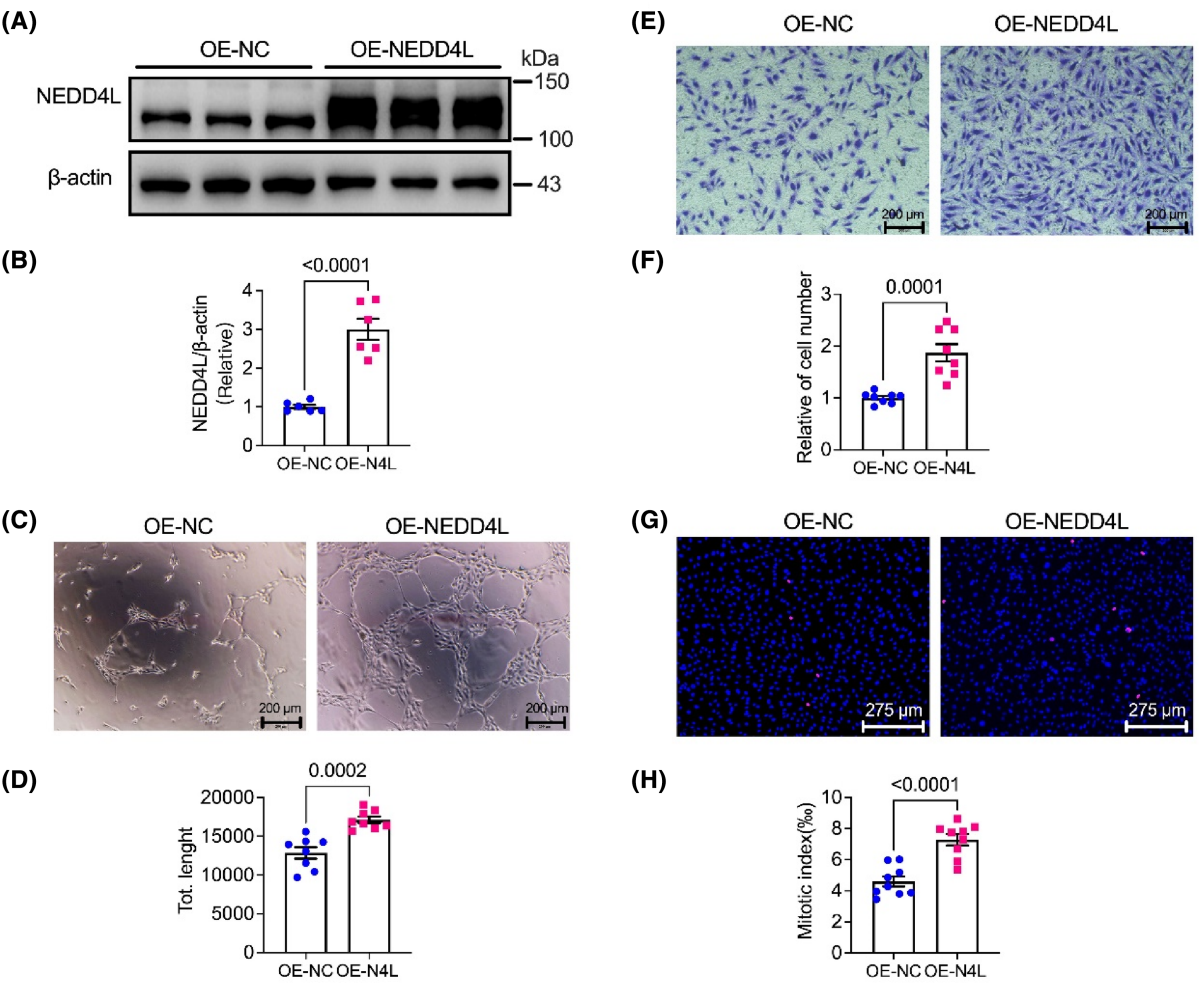
Overexpression of NEDD4L promoted angiogenesis, cell proliferation and migration. (A) Representative western blotting images of NEDD4L and β‐actin protein level in HUVECs infected with control adenovirus (OE‐NC) and NEDD4L overexpression adenovirus (OE‐NEDD4L). (B) Data analysis of relative expression of NEDD4L/β‐actin (*n* = 6 in each group). (C) Representative tube formation images with Matrigel matrix assay in HUVECs infected with control adenovirus (OE‐NC) and NEDD4L overexpression adenovirus (OE‐NEDD4L). (D) Analysis of tube formation with ImageJ software revealed the total length (*n* = 8 in each group). (E) Representative transwell images of HUVECs infected with control adenovirus (OE‐NC) and NEDD4L overexpression adenovirus (OE‐NEDD4L). (F) Data analysis of migrated cells in the transwell assay (*n* = 8 in each group). (G) Representative Phospho‐Histone H3 immunostaining images of HUVECs infected with control adenovirus (OE‐NC) and NEDD4L overexpression adenovirus (OE‐NEDD4L). (H) Data analysis of proliferated cells in the Phospho‐Histone H3 staining (*n* = 9 in each group).

We next established the role of overexpression NEDD4L on HUVECs function by infected with adenovirus which carried the NEDD4L overexpressed vector. The expression of NEDD4L in control adenovirus (OE‐NC) and NEDD4L overexpression adenovirus (OE‐NEDD4L) infected HUVECs was detected, and showed a significant promotion with about 3‐fold in the NEDD4L overexpression adenovirus‐infected cells compared to the control (Figure 2A,B). The tube formation, cell migration and proliferation in NEDD4L‐overexpressed HUVECs were also tested. Intriguingly, the angiogenic effect of OE‐NEDD4L was promoted (Figure 2C,D), and the migrated cells (Figure 2E,F) and the pHH3‐positive cells (Figure 2G,H) were significantly increased by OE‐NEDD4L compared to control. These results implied an important role of NEDD4L on the angiogenic effect in HUVECs.

### 3.3 Knockdown of NEDD4L restrained the phosphorylation of Akt, Erk1/2 and eNOS and the expression of VEGFR2 and Cyclin D1 and D3

**FIGURE 3:**
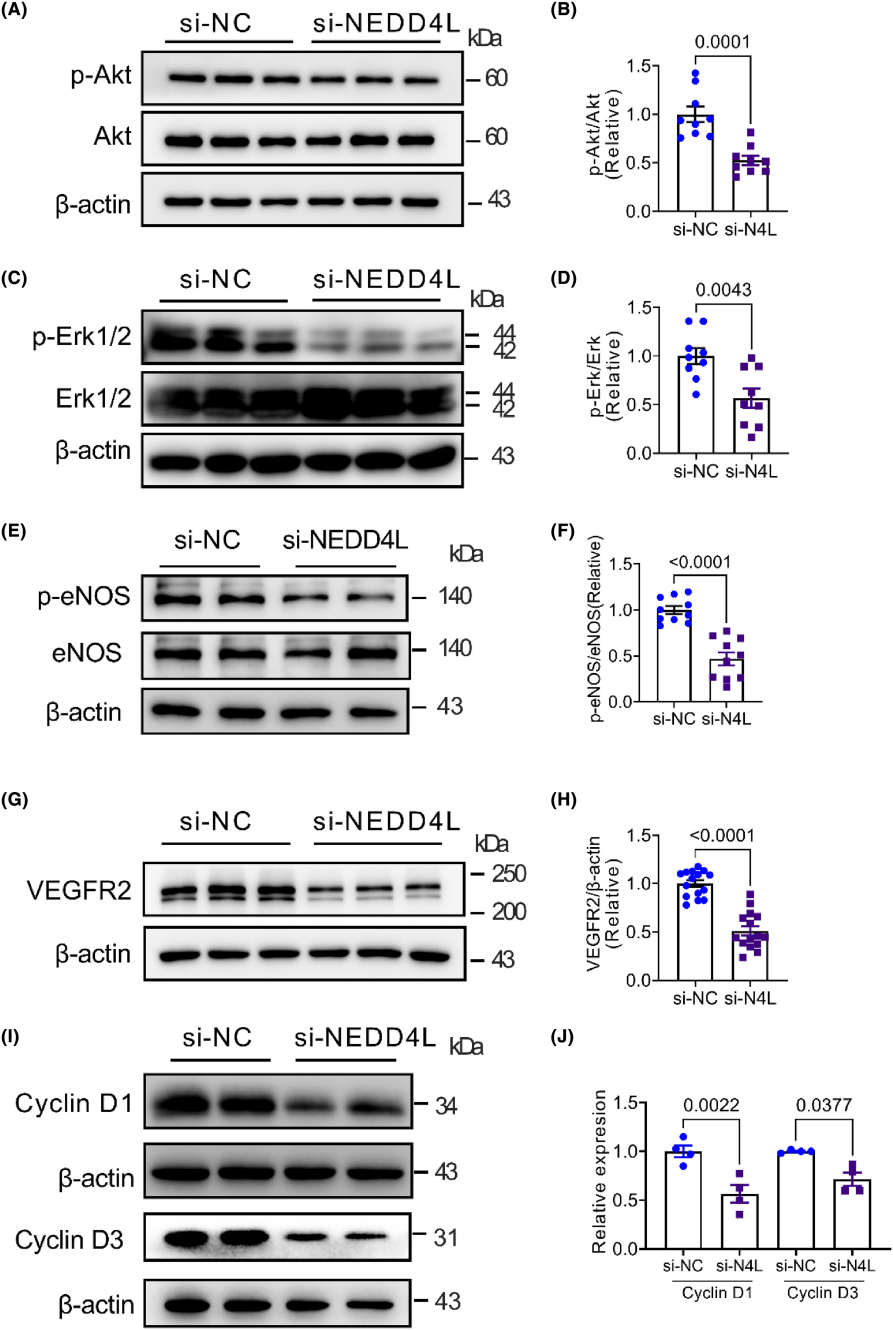
Molecular pathway in NEDD4L deficient HUVECs. (A) Representative western blotting images of phospho‐Akt (p‐Akt), Akt and β‐actin in HUVECs transfected with control siRNA (si‐NC) or NEDD4L siRNA (si‐NEDD4L). (B) Data analysis of relative expression of p‐Akt/Akt (*n* = 9 in each group). (C) Representative western blotting images of phospho‐Erk1/2 (p‐Erk1/2), Erk1/2 and β‐actin in HUVECs transfected with control siRNA (si‐NC) or NEDD4L siRNA (si‐NEDD4L). (D) Data analysis of relative expression of p‐Erk/Erk (*n* = 9 in each group). (E) Representative western blotting images of phospho‐eNOS and eNOS in HUVECs transfected with control siRNA (si‐NC) or NEDD4L siRNA (si‐NEDD4L). (F) Data analysis of relative expression of p‐eNOS/eNOS (*n* = 10 in each group). (G) Representative western blotting images of VEGFR2 and β‐actin in HUVECs transfected with control siRNA (si‐NC) or NEDD4L siRNA (si‐NEDD4L). (H) Data analysis of relative expression of VEGFR2/β‐actin (*n* = 15 in each group). (I) Representative western blotting images of Cyclin D1, Cyclin D3 and β‐actin in HUVECs transfected with control siRNA (si‐NC) or NEDD4L siRNA (si‐NEDD4L). (J) Data analysis of relative expression of Cyclin D1/β‐actin and Cyclin D3/β‐actin (*n* = 4 in each group).

To determine the signal molecules involved in NEDD4L‐regulated cell proliferation and angiogenic activity, the proliferation‐related molecules were determined by western blot assay in control and NEDD4L siRNA‐transfected HUVECs. Figure 3 illustrates the impact of the knockdown of NEDD4L on survival kinase Akt (Ser473), the mitogen‐activated protein kinase P42/44 (Erk1/2), endothelial nitric oxide synthase (eNOS), VEGFR2 and the cell cycle‐related proteins cyclin D1 and D3 in HUVECs. It is interesting to note that phosphorylation of Akt (Ser473) (Figure 3A,B), Erk1/2 (Figure 3C,D) and eNOS (Figure 3E,F) and the expression of VEGFR2 (Figure 3G,H), and cyclin D1 and D3 (Figure 3I,J) were significantly decreased in NEDD4L deficient HUVECs compared to the control group.

### 3.4 Overexpression of NEDD4L promoted the phosphorylation of Akt, Erk1/2 and eNOS and the expression of VEGFR2 and Cyclin D1 and D3

**FIGURE 4:**
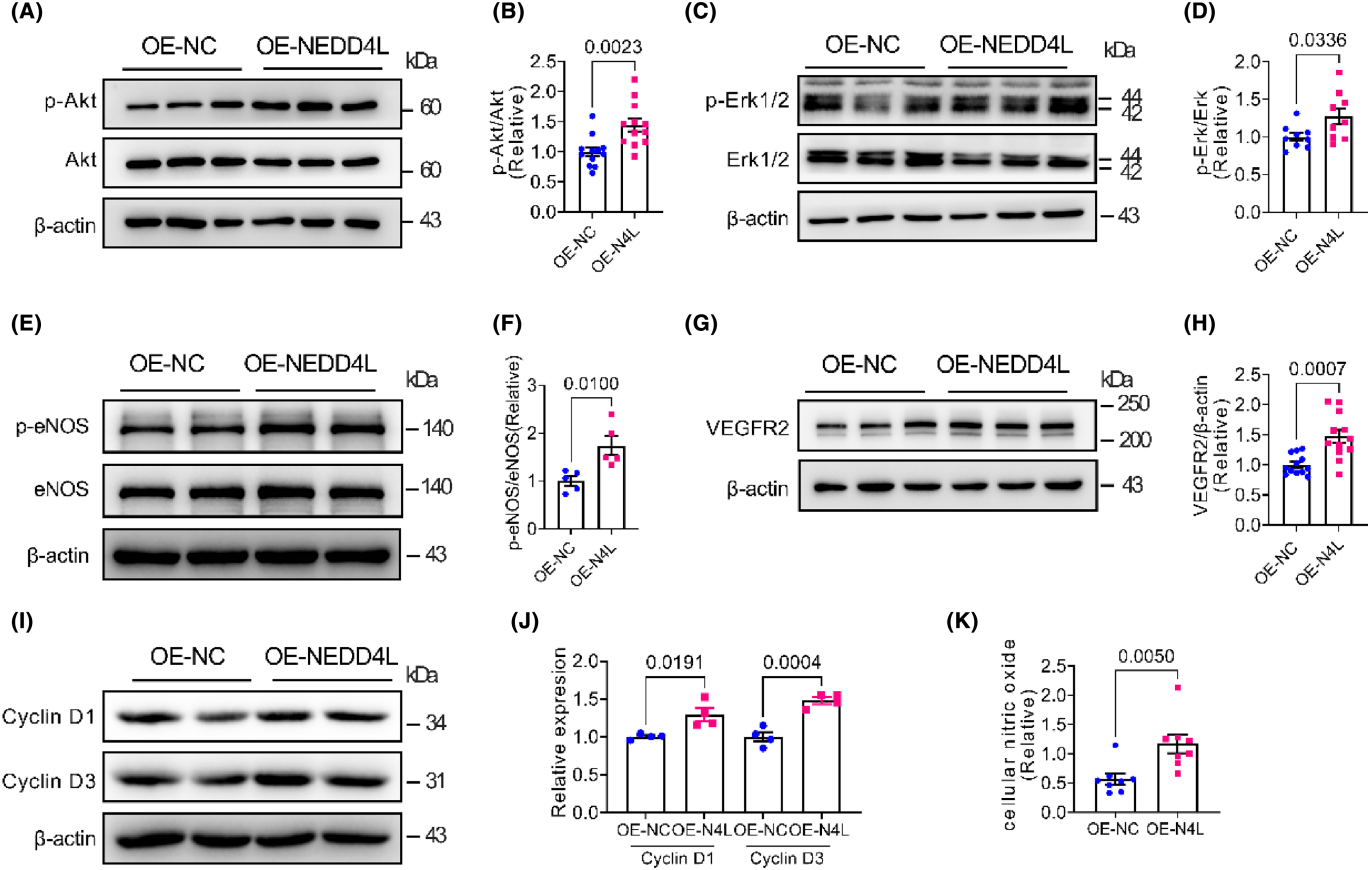
NEDD4L overexpression regulated pathway. (A) Representative western blotting images of phospho‐Akt (p‐Akt), Akt and β‐actin in HUVECs infected with control adenovirus (OE‐NC) or NEDD4L overexpression adenovirus (OE‐NEDD4L). (B) Data analysis of relative expression of p‐Akt/Akt (*n* = 15 in each group). (C) Representative western blotting images of phospho‐Erk1/2 (p‐Erk1/2), Erk1/2 and β‐actin in HUVECs infected with control adenovirus (OE‐NC) or NEDD4L overexpression adenovirus (OE‐NEDD4L). (D) Data analysis of relative expression of p‐Erk/Erk (*n* = 9 in each group). (E) Representative western blotting images of phospho‐eNOS (p‐eNOS) and eNOS in HUVECs infected with control adenovirus (OE‐NC) or NEDD4L overexpression adenovirus (OE‐NEDD4L). (F) Data analysis of relative expression of p‐eNOS/eNOS (*n* = 5 in each group). (G) Representative western blotting images of VEGFR2 and β‐actin in HUVECs infected with control adenovirus (OE‐NC) or NEDD4L overexpression adenovirus (OE‐NEDD4L). (H) Data analysis of relative expression of VEGFR2/β‐actin (*n* = 12 in each group). (I) Representative western blotting images of Cyclin D1, Cyclin D3 and β‐actin in HUVECs infected with control adenovirus (OE‐NC) or NEDD4L overexpression adenovirus (OE‐NEDD4L). (J) Data analysis of relative expression of Cyclin D1/β‐actin and Cyclin D3/β‐actin (*n* = 4 in each group). (K) Analysis of intracellular concentration of nitric oxide (NO) in HUVECs infected with control adenovirus (OE‐NC) or NEDD4L overexpression adenovirus (OE‐NEDD4L) (*n* = 8 in each group).

We next examined the phosphorylation of Akt, Erk1/2 and eNOS and the expression of VEGFR2 and cyclin D1 and D3 in control adenovirus and NEDD4L overexpression adenovirus‐infected HUVECs. Overexpression of NEDD4L exhibited a promotion effect on these proteins, such as promoting the phosphorylation of Akt (Figure 4A,B), Erk1/2 (Figure 4C,D) and eNOS (Figure 4E,F), and increasing the expression of VEGF2 (Figure 4G,H) and cyclin D1 and D3 (Figure 4I,J). Moreover, the cellular nitric oxide (NO) concentration was also promoted by overexpression of NEDD4L (Figure 4K).

### 3.5 Pathway inhibitors suppressed the pro‐angiogenic effect of NEDD4L

**FIGURE 5:**
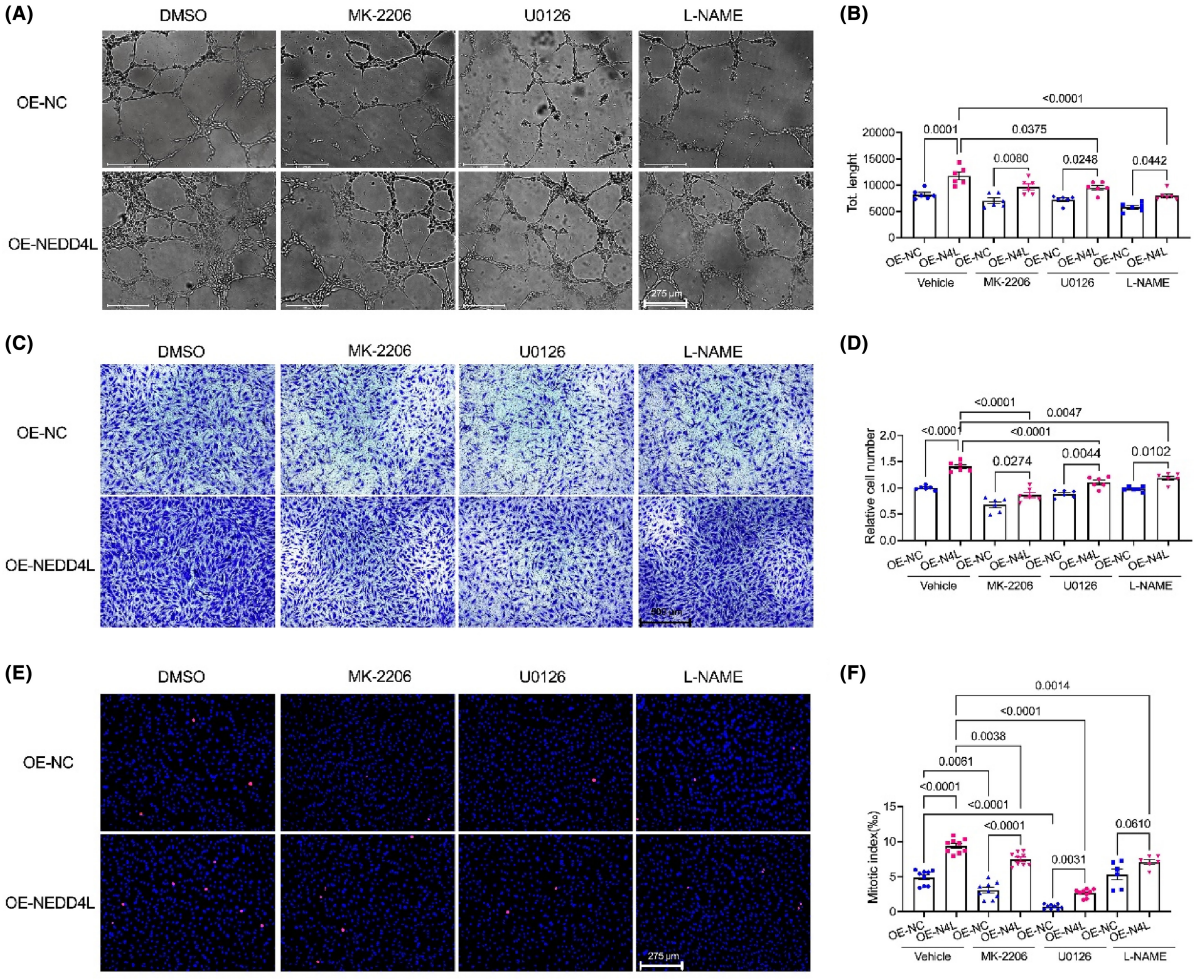
Inhibition of Akt, Erk1/2 and eNOS pathways suppressed NEDD4L‐promoted angiogenesis, cell proliferation and migration. (A) Representative tube formation images with Matrigel matrix assay in HUVECs treated with vehicle (DMSO), Akt blocker MK‐2206, Erk1/2 blocker U0126 and eNOS blocker L‐NAME after infected with control adenovirus (OE‐NC) and NEDD4L overexpression adenovirus (OE‐NEDD4L). (B) Analysis of tube formation with ImageJ software revealed the total length (*n* = 6 in each group). (C) Representative transwell images in HUVECs treated with vehicle (DMSO), Akt blocker MK‐2206, Erk1/2 blocker U0126 and eNOS blocker L‐NAME after infected with control adenovirus (OE‐NC) and NEDD4L overexpression adenovirus (OE‐NEDD4L). (D) Data analysis of migrated cells in the transwell assay (*n* = 6 in each group). (E) Representative pHH3 immunostaining images in HUVECs treated with vehicle (DMSO), Akt blocker MK‐2206, Erk1/2 blocker U0126 and eNOS blocker L‐NAME after infected with control adenovirus (OE‐NC) and NEDD4L overexpression adenovirus (OE‐NEDD4L). (F) Data analysis of proliferated cells in the pHH3 staining (*n* = 9 in each group).

To further examine the molecular pathway in NEDD4L‐regulated angiogenic effect, we employed the Akt inhibitor MK‐2206, Erk1/2 inhibitor U0126 and eNOS inhibitor L‐NAME in control or NEDD4L overexpressed HUVECs. Figure 5A,B shows that NEDD4L overexpression‐promoted tube formation was significantly suppressed by MK‐2206, U0126 and L‐NAME. Consistent with this effect, these inhibitors also suppressed the migrating cells (Figure 5C,D) and proliferating cells (Figure 5E,F) in NEDD4L overexpressed HUVECs. These results implied that the NEDD4L‐regulated angiogenic effect was mediated by the Akt/Erk1/2/eNOS pathway.

### 3.6 Inhibition of the Akt/Erk1/2/eNOS pathway restrained the NEDD4L‐promoted cyclin D1 and D3

**FIGURE 6:**
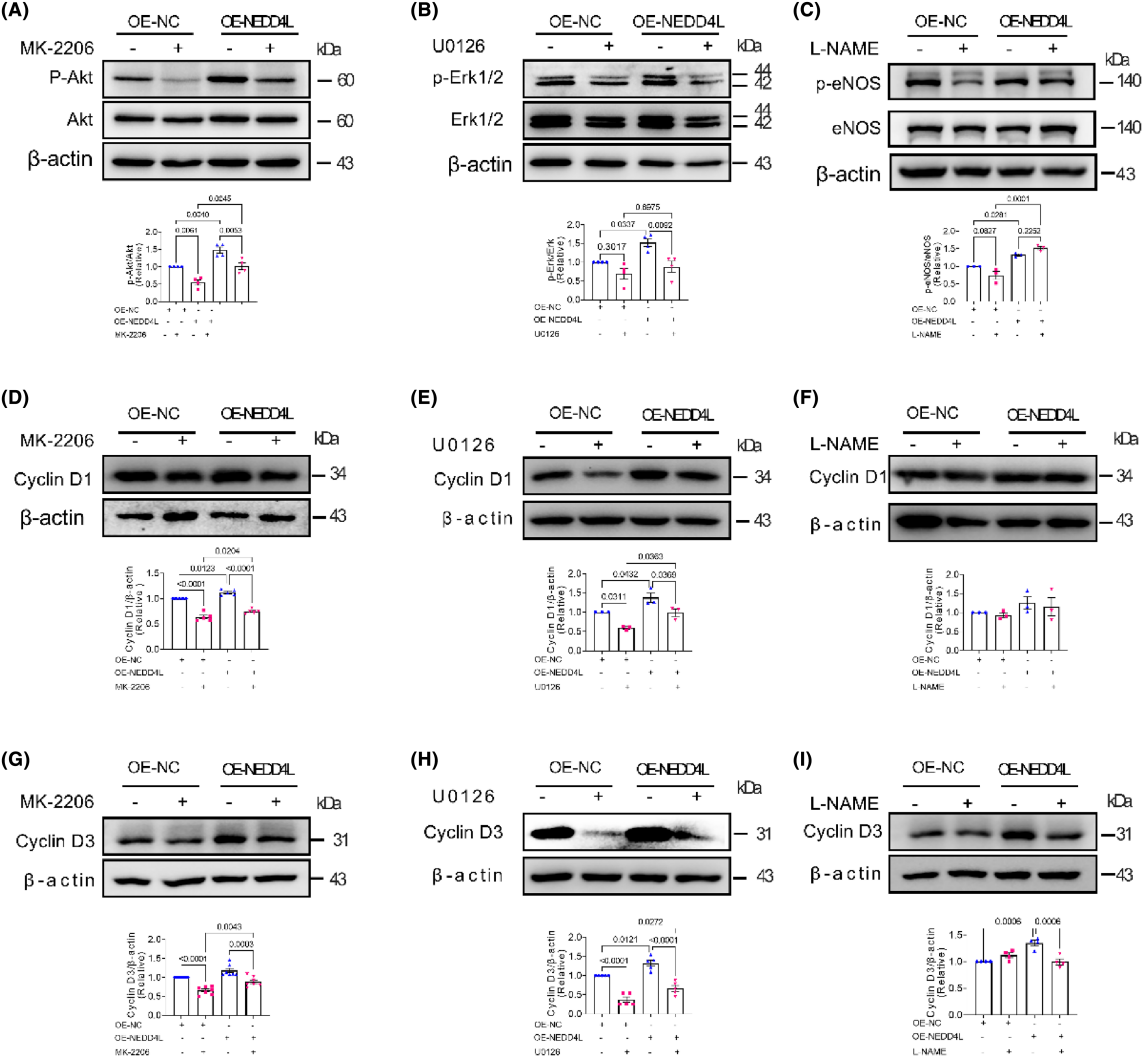
Signalling pathway blockers restrained NEDD4L‐regulated Akt/Erk1/2/eNOS pathway. (A) Representative western blotting images and data analysis of relative expression of phosphor‐Akt (p‐Akt), Akt and β‐actin in HUVECs treated with vehicle (DMSO) and Akt blocker MK‐2206 after infected with control adenovirus (OE‐NC) and NEDD4L overexpression adenovirus (OE‐NEDD4L) (*n* = 4 in each group). (B) Representative western blotting images and data analysis of relative expression of phospho‐Erk1/2, Erk1/2 and β‐actin in HUVECs treated with vehicle (DMSO) and Erk1/2 blocker U0126 after infected with control adenovirus (OE‐NC) and NEDD4L overexpression adenovirus (OE‐NEDD4L) (*n* = 4 in each group). (C) Representative western blotting images and data analysis of relative expression of p‐eNOS and eNOS in HUVECs treated with vehicle (DMSO) and eNOS blocker L‐NAME after infected with control adenovirus (OE‐NC) and NEDD4L overexpression adenovirus (OE‐NEDD4L) (*n* = 4 in each group). Representative western blotting images and data analysis of relative expression of Cyclin D1 and β‐actin in HUVECs treated with Akt blocker MK‐2206 (*n* = 5 in each group) (D), Erk1/2 blocker U0126 (*n* = 3 in each group) (E) and eNOS blocker L‐NAME (*n* = 3 in each group) (F) after infected with control adenovirus (OE‐NC) and NEDD4L overexpression adenovirus (OE‐NEDD4L). Representative western blotting images and data analysis of relative expression of Cyclin D3 and β‐actin in HUVECs treated with Akt blocker MK‐2206 (*n* = 7 in each group) (G), Erk1/2 blocker U0126 (*n* = 5 in each group) (H) and eNOS blocker L‐NAME (*n* = 4 in each group) (I) after infected with control adenovirus (OE‐NC) and NEDD4L overexpression adenovirus (OE‐NEDD4L).

We then determined the overexpression NEDD4L‐regulated molecular pathway detected by western blotting using MK‐2206, U0126 and L‐NAME. NEDD4L‐promoted phosphorylation of Akt was significantly suppressed by MK‐2206 (Figure 6A). NEDD4L‐promoted phosphorylation of Erk1/2 was restrained by U0126 (Figure 6B), and the phosphorylation of eNOS was decreased by L‐NAME (Figure 6C). Moreover, NEDD4L‐promoted expression of cyclin D1 was also being suppressed by MK‐2206 and U0126 (Figure 6D,E). However, the eNOS blocker L‐NAME does not have a restraining effect on NEDD4L‐promoted cyclin D1 (Figure 6F). Similarly, the expression of cyclin D3 was also restrained by MK‐2206 (Figure 6G), U0126 (Figure 6H) and L‐NAME (Figure 6I). The above data implied that NEDD4L‐promoted angiogenic effect was regulated by the Akt/ Erk1/2/eNOS pathway.

## 4 DISCUSSION

The E3 ubiquitin ligase NEDD4L is involved in a wide range of regulatory physiology and pathology conditions, such as hypertension, cardiovascular disease and tumorigenesis. However, the connections between NEDD4L and angiogenesis have yet to be well understood. Our findings demonstrated the critical role of NEDD4L in regulating endothelial cell progress, including angiogenesis, cell migration and proliferation. We reported that deficiency of NEDD4L suppressed angiogenesis, cell proliferation and migration, whereas overexpression promoted these functions. This intriguing finding of NEDD4L‐regulated angiogenesis and cell proliferation was regulated by the Akt/Erk1/2/eNOS pathways.

Accumulating evidence shows that NEDD4L is involved in the formation and development of hypertension at the molecular level by regulating sodium homeostasis and also participates in various cardiovascular diseases.^17^, ^19^, ^26^, ^27^, ^28^, ^29^ Our previous study has demonstrated that NEDD4L is involved in miR‐30a‐5p‐attenuated atherosclerosis by regulating macrophage polarization and lipid metabolism.^21^ However, the role of NEDD4L in angiogenesis was not clearly explored in HUVECs. In our results, the knockdown of NEDD4L inhibits cell proliferation and migration as well as the angiogenic effect in HUVECs. Interestingly, overexpression of NEDD4L promoted these functions. Nevertheless, in a previous study on oral squamous cell carcinoma, NEDD4L overexpression suppressed proliferation, cell cycle transition and glycolysis and inhibited in vivo tumour growth.^30^ In prostate cancer cells, NEDD4L overexpression reduced its proliferation.^31^ In addition, NEDD4L participated in JP1 (18‐F‐NFP‐JP1)‐inhibited melanoma growth and metastasis and prolonged the survival of mouse^32^ and ALCAP2 (β, β‐dimethyl‐acryl‐alkannin)‐suppressed lung adenocarcinoma cell proliferation, migration and invasion.^33^ NEDD4L inhibits bladder cancer progression by inactivating the p62/Keap1/Nrf2 pathway.^34^ Although these reports showed a conflicting conclusion of NEDD4L on cell proliferation or migration, we speculated that the NEDD4L may exert different roles in different cells.

Regulation of PI3K/Akt signalling has emerged as a critical component for effective angiogenesis.^35^ A series of studies have proved that PI3K/Akt signalling is regulated by NEDD4L in different cancer cells. Mutations of NEDD4L lead to Akt–mTOR pathway deregulation and cause periventricular nodular heterotopia.^36^ NEDD4L catalyses PIK3CA polyubiquitination, leading to its proteasome‐dependent degradation.^37^ NEDD4L inhibited lung adenocarcinoma cell progression in vitro and in vivo via inducing the ubiquitination‐mediated Ubiquitin‐conjugating enzyme E2T (UBE2T) degradation, which repressed PI3K‐Akt signalling.^38^ However, no evidence has reported the role of NEDD4L on the regulation of PI3K/Akt signalling in endothelial cells. In our present study, NEDD4L‐mediated cell proliferation, migration and angiogenesis were regulated by the Akt pathway in HUVECs, which is validated by Akt inhibitor MK‐2206. Furthermore, in hepatocellular carcinoma, downregulation of NEDD4L promotes tumour growth and inhibits MAPK/Erk1/2 signal pathway.^39^ Intriguingly, this effect of NEDD4L on MAPK/Erk1/2 is consistent with our present study in HUVECs.

Endothelial nitric oxide (NO) is a critical mediator of vascular function and vascular remodelling.^40^ NO is produced by endothelial nitric oxide synthase (eNOS). Loss of NO is a hallmark of endothelial dysfunction, promoting the pathophysiology of hypertension and atherosclerosis.^41^ In this study, deficiency of NEDD4L prevented the phosphorylation of eNOS, indicating an important role of NEDD4L on endothelial cell function. Additionally, Akt activates endothelial nitric oxide synthase (eNOS) that could be produced by endothelial cells, participates in the regulation of cell permeability,^40^ reflects the endothelial activity and maintains endothelial function.^42^ Our data have shown that overexpression of NEDD4L‐promoted Akt also increases the phosphorylation of eNOS.

There are some limitations in the present study. First, the NEDD4L‐regulated angiogenic effect was not validated in pathological conditions in animal models, such as hind‐limb ischemia and ischemic heart disease. The second limitation is that further and deeper mechanisms have yet to be fully explored in the NEDD4L‐regulated molecular pathway. In addition, to improve these limitations, we will explore the role of NEDD4L in regulating hind‐limb and myocardial infarction using endothelial cell‐specific knock‐out or knock‐in mice in our future study. Moreover, because NEDD4L is a ubiquitin ligase, we will also screen and find the ubiquitin targets of NEDD4L in regulating angiogenesis. The third limitation is that we have not selected a specific activator of NEDD4L for treating ischemic disease, such as myocardial infarction, diabetes‐related limb ischemia.

In summary, our present study has demonstrated novel evidence that NEDD4L is a critical mediator in promoting angiogenesis in HUVECs. Mechanistically, we revealed that NEDD4L directly regulated the phosphorylation Akt, Erk1/2 and eNOS and upregulated the expression of cyclin D1 and D3. The NEDD4L/Akt/Erk/Cyclin D1/3 axis is critical for regulating angiogenesis. In addition, pharmacological or genetic promotion of NEDD4L may represent a promising therapeutic strategy for treating ischemic diseases, such as myocardial infarction, exacerbates diabetic retinopathy and critical limb ischemia, by promoting angiogenic role in the ischemic tissues.

## AUTHOR CONTRIBUTIONS

**Binghong Liu:** Conceptualization (equal); data curation (lead); formal analysis (lead); investigation (lead); methodology (lead); project administration (lead); software (lead); validation (lead); visualization (lead); writing – review and editing (supporting). **Fei Song:** Formal analysis (supporting); methodology (supporting). **Xiaoxia Zhou:** Formal analysis (supporting); methodology (supporting). **Chan Wu:** Methodology (supporting). **Huizhu Huang:** Methodology (supporting). **Weiyin Wu:** Methodology (supporting). **Gang Li:** Conceptualization (lead); funding acquisition (equal); supervision (lead); visualization (equal); writing – original draft (lead); writing – review and editing (lead). **Yan Wang:** Conceptualization (lead); funding acquisition (lead); supervision (lead); writing – original draft (equal); writing – review and editing (lead).

## FUNDING INFORMATION

This study was in part supported by the National Natural Science Foundation of China (Grant No. 81970283).

## CONFLICT OF INTEREST STATEMENT

The authors declare no competing interests.

## Data Availability

The data that support the findings of this study are available from the corresponding author upon reasonable request.
